# Nucleotide Metabolism Behind Epigenetics

**DOI:** 10.3389/fendo.2021.731648

**Published:** 2021-08-30

**Authors:** Tamaki Suganuma, Jerry L. Workman

**Affiliations:** Workman Lab Stowers Institute for Medical Research, Kansas City, MO, United States

**Keywords:** nucleotide metabolism, chromatin modifiers, DNA damage, metabolism, histone modifications, NAD, ADP-ribosylation, RNA editing

## Abstract

The mechanisms of epigenetic gene regulation—histone modifications, chromatin remodeling, DNA methylation, and noncoding RNA—use metabolites as enzymatic cofactors and substrates in reactions that allow chromatin formation, nucleotide biogenesis, transcription, RNA processing, and translation. Gene expression responds to demands from cellular processes that use specific metabolites and alters or maintains cellular metabolic status. However, the roles of metabolites—particularly nucleotides—as regulatory molecules in epigenetic regulation and biological processes remain largely unknown. Here we review the crosstalk between gene expression, nucleotide metabolism, and cellular processes, and explore the role of metabolism in epigenetics as a critical regulator of biological events.

## Introduction

One-carbon metabolism governs cellular nutritional status by sensing input metabolites and generating and redistributing output metabolites (1, 2). The input metabolites of one-carbon metabolism are usually serine and glycine reacting in the folate cycle; output metabolites include S-adenosyl-L-methionine (SAMe), glutathione, nucleotides, and polyamines. This one-carbon metabolic network generates energy and coordinates intracellular redox status. One-carbon metabolism has been targeted in cancer therapy (1, 3), for example by using pyrimidine analogues such as fluorouracil, which inhibits thymidylate synthase in the folate metabolic pathway (3). Outputs of one-carbon metabolism play key roles in post-translational modification of histones, DNA methylation, chromatin remodeling, and RNA biogenesis including tRNA modifications (2, 4).

Chromatin modifications regulate transcription and gene expression to modify or maintain the cellular status. Metabolites are essential cofactors and substrates in the epigenetic marking of histones including those in nucleosomes. For example, histone/lysine acetyltransferases use acetyl-CoA to catalyze the addition of an acetyl group to an epsilon amino group of a lysine sidechain in the N-terminal “tail” domains of histones (5). Lysine acylation reactions also include propionylation, butyrylation, crotonylation, β-hydroxybutyrylation, succinylation, malonylation, and glutarylation (6). These different acylation reactions use different short-chain acyl intermediates, suggesting that the type of acylation is determined by the cell metabolic status. Histone deacetylases (HDACs) catalyze the hydrolysis of an *N*-ε-acetyl-L-lysine sidechain to generate acetate and L-lysine (7). Class I and II HDACs catalyze this hydrolysis in a Zn^+^-dependent manner; Class III HDACs (sirtuins) use the oxidized form of nicotinamide adenine dinucleotide, NAD^+^, as a co-substrate and transfer the acetyl group onto adenosine diphosphate ribose (ADPR) to form a 2’-O-acetyl-ADPR (8, 9). Therefore, sirtuins may be sensitive to cellular NAD/energy levels in their regulation of transcription. Methylation of histones and DNA requires SAMe (which is synthesized from ATP and methionine by methionine adenosyltransferase) as a cofactor. Histone demethylations are catalyzed by two major classes of lysine demethylases: lysine-specific histone demethylases (LSDs) and JmjC-domain-containing histone demethylases (JMJCs) (2). LSDs require flavin adenine dinucleotide (FAD), a redox-active coenzyme that is synthesized *de novo* from riboflavin (vitamin B2) (10). Demethylation by JMJCs requires Fe(II), consumes α-ketoglutaric acid/2-oxoglutaric acid and O_2,_ and produces succinate, CO_2_, and formaldehyde (11); these demethylation reactions are directly involved in redox reactions. These reversible histone modifications enable crosstalk of metabolism with epigenetic regulation *via* coordination of nucleosome modifiers (12).

Nucleosome modifiers may sense cellular status by using metabolites in reactions that regulate gene expression. For example, acetylation and methylation can occur on the same lysine substrate, such as in histone H3 lysine 9 (H3K9) methylation and acetylation in mammals. H3K9 methylation is seen in silenced chromatin; however, H3K9 acetylation is a mark for transcription activity. Notably, a nontranscriptional function of histone methylation plays an important role in mediating gene expression and metabolism in *Saccharomyces cerevisiae*. Excess methyl group formed by abolishing phospholipid methylation is stored in core histones, leading to increased H3K36, K79, and K4 methylation (13). This feature of histones as methyl sinks adjusts the intracellular levels of toxic sulfides and reactive oxygen species (ROS) which are regulated in transsulfuration and sulfur amino acid catabolism (13). Metabolic enzymes not only catalyze metabolite generation, but also participate in chromatin modification. For example, in *S. cerevisiae*, Pyk1, a homolog of human pyruvate kinase M2 (PKM2), is a component of the serine-responsive SAM-containing metabolic enzyme (SESAME) complex, which contains serine metabolic enzymes (Ser33, Ser3, and Shm2), SAMe synthetases (Sam1 and Sam2), and an acetyl-CoA synthase (Acs2) (14). Pyk1 in SESAME phosphorylates H3T11 by utilizing phosphoenolpyruvate as the phosphate donor instead of ATP (14). The interaction of SESAME with the Set1 H3K4 methyltransferase complex enables crosstalk between H3K4 methylation and H3T11 phosphorylation in response to glycolysis and glucose-dependent serine metabolism (14). Remarkably, loss of phosphorylation on H3T11 extends the chronological lifespan of *S. cerevisiae* (15). Thus, histone and chromatin modifications use metabolites to regulate gene expression and cellular metabolism.

An important production of one-carbon metabolism is nucleotide biosynthesis. Nucleotides are fundamental components of nucleosomes and RNA transcripts. However, it remains unclear how nucleotide/nucleoside metabolism influences chromatin modifications and epigenetics. Therefore, in this review we focus our discussion on nucleotide metabolism in epigenetic regulation and biological processes.

## Chromatin Modifiers and Nucleotide Biosynthesis

Transcription is a mechanism to regulate metabolism and is regulated by chromatin modifiers. Chromatin modifiers often form protein complexes that modify chromatin structure and/or place epigenetic marks. ATP-dependent chromatin remodeling by the Switch/sucrose non-fermentable (SWI/SNF) family plays important function in gene activation and displaces acetylated histones (12). BAF60a, a subunit of SWI/SNF promotes chromatin accessibility at PPARγ and EBF2 transcription factor binding sites on thermogenic genes in brown and beige adipogenesis and thermogenesis (16). However, adipocyte-specific BAF60a knockout mice show more pronounced cold-induced browning of inguinal white adipose tissue induced by MC2R, a receptor for the adrenocorticotropic hormone (16). Thus, unexplored roles of SWI/SNF in energy generation is under consideration.

**Figure 1:**
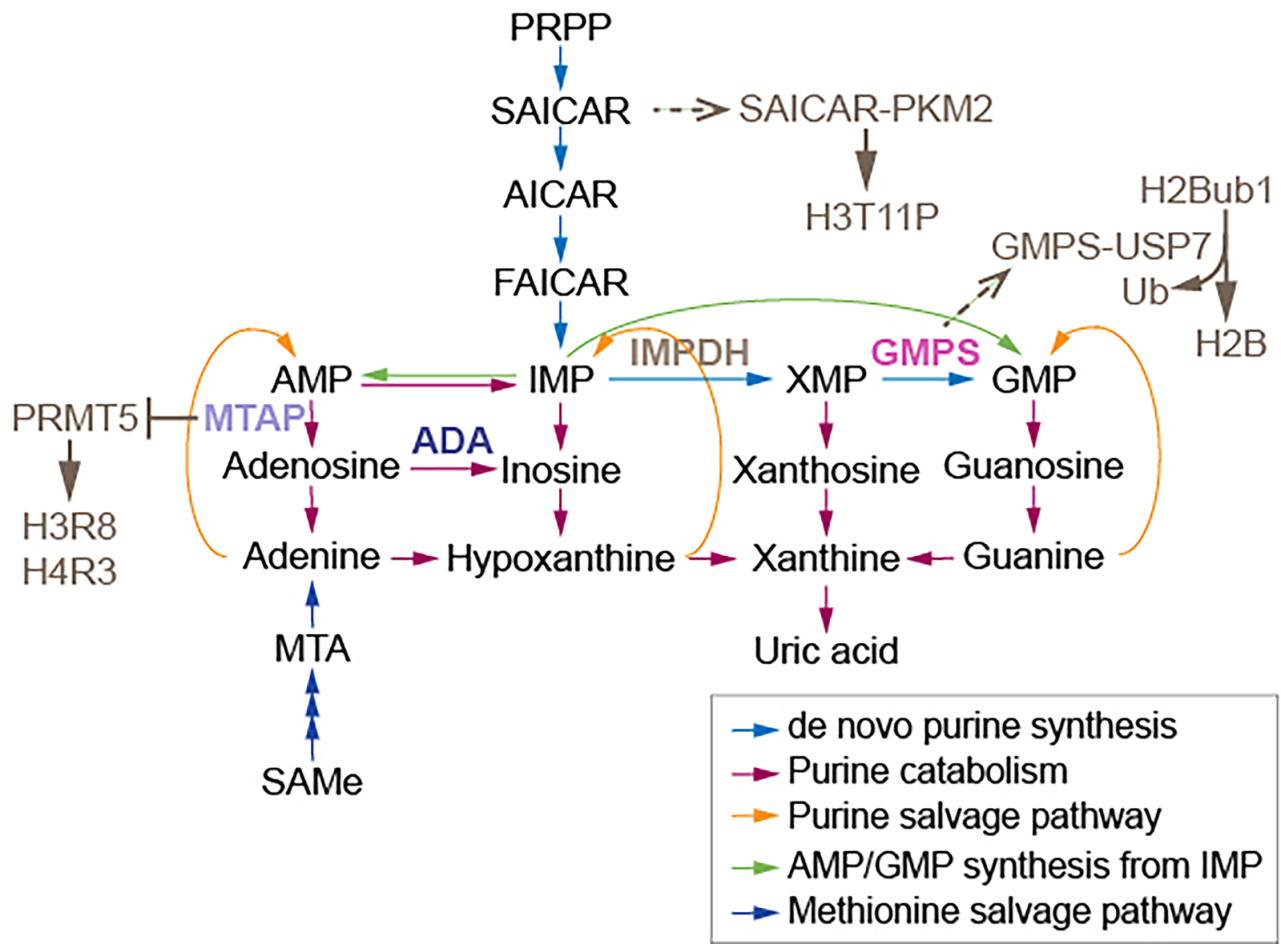
Enzymes and a synthase relaying on purine metabolism modify nucleosomes. The association of GMPS with USP7 removes monoubiquitylation of histone H2B. The association of SAICAR with PKM2 facilitates H3T11 phosphorylation. MTAP suppresses PRMT5, which methylates H3 arginine (R) 8 and H4R3. AMP, adenosine monophosphate; MTA, 5’-methylthioadenosine; PRPP, 5-phosphoribosyl-1-pyrophosphate; AICAR, 5-amino-1-[3,4-dihydroxy-5-(hydroxymethyl)oxolan-2-yl]imidazole-4-carboxamide; FAICAR, 5-formamidoimidazole-4-carboxamide ribotide; IMP, inosine monophosphate; XMP, xanthosine monophosphate; GMP, guanosine monophosphate; IMPDH, inosine monophosphate dehydrogenase; GMPS, guanine monophosphate synthase. Figure adapted from (21).

**Figure 2:**
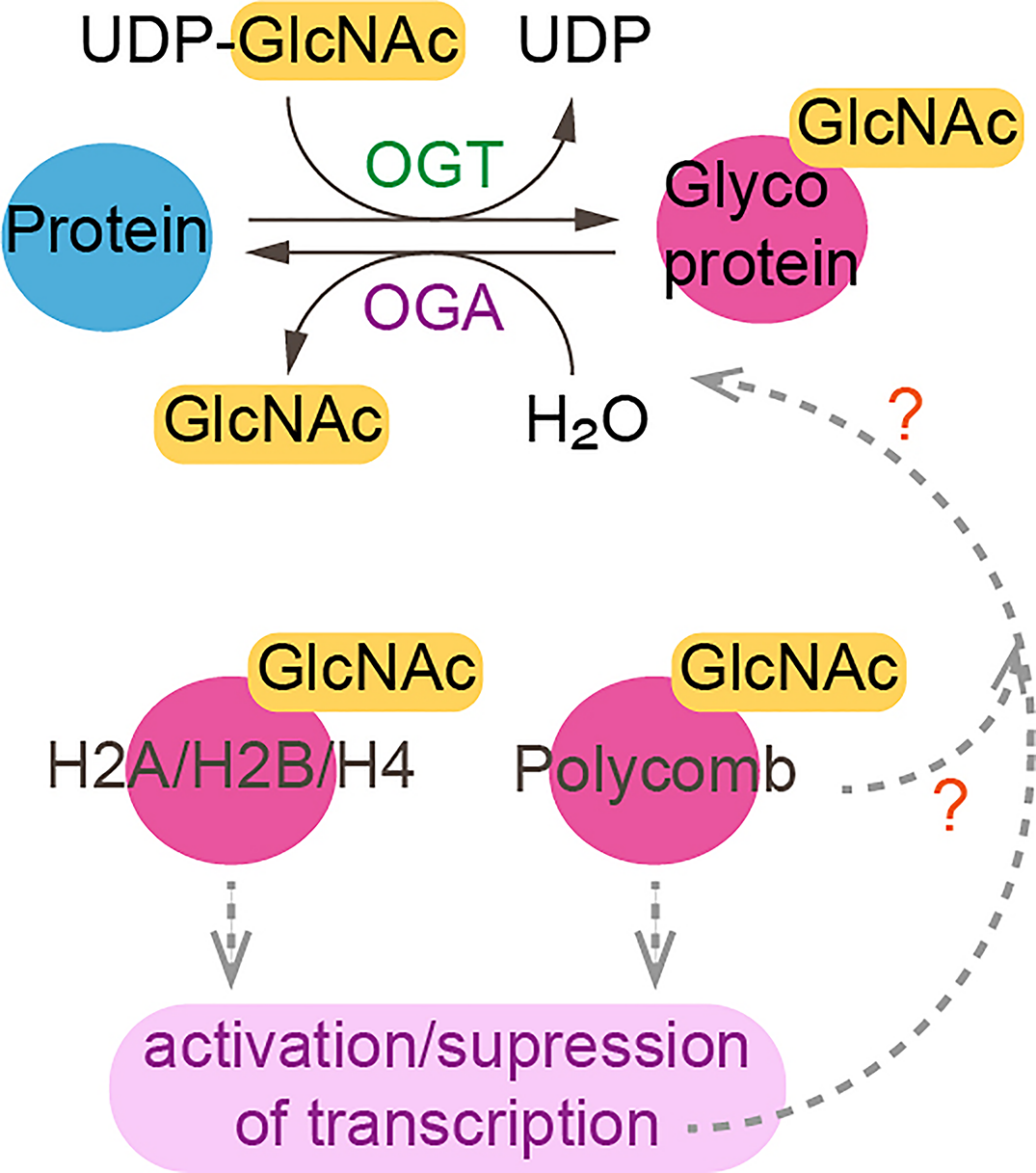
*O*-linked-N-acetylglucosaminylation. *O*-linked-N-acetylglucosaminylation (*O*-GlcNAcylation) occurs when *O*-GlcNAc is added to serine or threonine residues of nuclear or cytoplasmic proteins by *O*-GlcNAc transferase (OGT) (24). This reaction is reversible as *O*-GlcNAc can be removed by *O*-GlcNAcase (OGA). Linkage of GlcNac to histones and polycomb subunits affects gene expression. ?, unknown.

Some chromatin modifiers contain nucleotide metabolic enzymes. In *Drosophila* and mammals, the nucleotide biosynthetic enzyme guanosine 5’-monophosphate synthase (GMPS) forms a heteromeric complex with ubiquitin protease 7 (USP7) (17, 18). This complex is recruited to polycomb response elements and promotes polycomb silencing of homeotic genes through methylation of histone H3K27 (17). The suggested mechanism is that USP7-GMPS removes ubiquitin from ubiquitylated H2B which is associated with active chromatin. In mammalian cells, GMPS promotes p53 stabilization in the presence of etoposide, which induces DNA damage (19). GMPS is critical for expression of the p53 target genes *p21* and *bax*, and for p53-dependent transcriptional suppression of *cdc6* and *mcm6* following etoposide treatment (19). GMPS also promotes *de novo* synthesis of guanine (20) (**Figure 1**). GMPS is highly expressed in ovary cancer tissue, in which p21 expression is reduced (22). A remaining question is whether recruitment of GMPS to the polycomb complex suppresses GMPS nucleoside synthase activity. Interestingly, the polycomb group gene super sex combs (*sxc*) encodes *O*-linked N-acetylglucosamine (GlcNAc) transferase (Ogt) in *Drosophila* (23). Ogt modifies serine and threonine residues of target proteins by adding a single N-acetylglucosamine in an *O*-glycosidic linkage (**Figure 2**). Lack of *sxc*/*Ogt* results in failure to maintain polycomb transcriptional repression. *O*-GlcNAcylation of polycomb complex subunits is necessary for proper targeting of *Hox* repression in human (25), the role of the polycomb complex in glycosylation is unknown (**Figure 2**). Monitoring the expression of polycomb target genes upon adding supplemental UDP to *sxc*/Ogt-null mutants may help address the question of whether polycomb suppresses its target genes by suppressing glycosylation or whether UDP is required as a cofactor for the repression of polycomb target genes.

## Nucleotides and DNA Damage

Nucleotide metabolic enzymes also have been found to directly modify nucleosomes. The interaction of SAICAR (phosphoribosylaminoimidazolesuccinocarboxamide) with PKM2 facilitates PKM2 activity and the phosphorylation and activation of Erk1/2, which is necessary for mitogen-induced cell proliferation in Hela cells (26, 27). SAICAR is an intermediate of *de novo* purine synthesis (**Figure 1**). SAICAR also stimulates histone H3T11 phosphorylation *in vitro* (27). Promotion of cell proliferation by SAICAR-PKM2 presumably involves cell cycle control and DNA replication because DNA replication is coordinated with nucleotide synthesis. Deoxynucleotide triphosphates (dNTPs) used in DNA replication are obtained from corresponding ribonucleotides through reduction reactions catalyzed by ribonucleotide reductase (RNR) (28). Imbalances in the dNTP pool increase mutation rates and replication anomalies; therefore, RNR activity and dNTP pool sizes are strictly regulated (29). At low ROS levels in mammalian cells, increased oligomerization of peroxiredoxin 2 (PRDX2) results in formation of a replisome-associated complex with the replication fork accelerator TIMELESS, which associates with chromatin (30). However, at high ROS levels induced by hydroxyurea inhibition of RNR, a smaller PRDX2 oligomer displaces TIMELESS from replisomes, resulting in attenuation of DNA replication (30). It remains unknown whether elevated ROS from different sources select different nucleotide synthetic pathways, and whether the pathways that generate ROS attenuate DNA replication.

Methylthioadenosine phosphorylase (MTAP) is required for the salvage of adenine and methionine (31) (**Figure 1**). Deletion of MTAP is frequently found in human tumors, including 53% of glioblastomas and 26% of pancreatic cancers (32). MTAP-deleted cancer cells accumulate methylthioadenosine (MTA), which inhibits the methyltransferase activity of protein arginine methyltransferase 5 (PRMT5) (32). The viability of MTAP-deleted cancer cells is diminished by depletion of PRMT5 (32), suggesting that lowed SAMe levels influence cancer cell viability, as PRMT5 is required for maintenance of cellular SAMe levels (33). It has been proposed that depletion of PRMT5 may be useful for cancer therapy (32); however, it is cautioned that PRMT5-dependent SAMe is required for proper mRNA splicing (33). It also remains unknown whether tumor suppressor genes, which may induce MTAP, are critically regulated by methyltransferase activity of PRMT5 in healthy cells. Inhibition of methionine adenosyltransferase 2A (MAT2A), which catalyzes the production of SAMe from ATP, reduces SAMe levels and inhibits the proliferation of MTAP-deleted HCT116 cells (34). However, MAT2A inhibition using AGI-24512 induces greater DNA damage in MTAP-deleted HCT116 cells than in wild type HCT116 cells (34). Another MAT2A inhibitor, AG-270, may be a stronger drug candidate than AGI-24512 as AG-270 reduces proliferation and DNA damage repair in MTAP-deleted cells (34). These observations suggest that loss of adenosine induces DNA damage in MTAP-deleted cells; however, no measurements of adenosine levels in MTAP-deleted cancer cells and HCT116 cells were performed. Such measurements of metabolites including nucleotides which require MTAP are needed to be understand the results of these preclinical studies of MAT2A inhibitors.

## Nicotinamide Adenine Dinucleotide

A metabolic cofactor consisting of nucleosides plays roles in redox reactions and in enzymatic reactions as a substrate. Nicotinamide adenine dinucleotide (NAD) is a central metabolic coenzyme due to its involvement in redox reactions. NAD consists of two nucleosides connected by a pyrophosphate. NAD exists in oxidized (NAD^+^) and reduced (NADH) forms. In mammalian cells, NAD^+^ is used by class III HDACs, including SIRT1 and SIRT2 (35). In yeast, Sir2 is homologous to human SIRT1 and functions in transcriptional silencing at the silent mating type loci HML and HMR and at telomeres as part of the SIR complex, which consists of Sir2, Sir3, and Sir4 (36). Sir2 deacetylates histones in an NAD^+^-dependent reaction. The deacetylation activity of Sir2 and other sirtuins is inhibited by nicotinamide, which inhibits silencing at the telomeres, mating type loci, and rDNA (37, 38). Mammalian SIRT1 and SIRT6 transcriptionally drive the circadian rhythm in distinct manners (39). Whereas SIRT1 establishes a repressive chromatin state by contributing to H3K9me2 and H3K27me3 at circadian clock-controlled genes by deacetylation of histone H3K9, BMAL1, and PER2 (40), SIRT6 regulates recruitment of another circadian activator, sterol regulatory element binding transcription factor 1 (SREBF1/SREBP1), to the promoter of the circadian clock-controlled gene *Fasn* (39). Removal of SIRT6 disrupts hepatic lipid metabolism and circadian rhythm (39). SIRT6 also regulates lysine fatty acylation, which removes long-chain fatty acyl group, such as myristoyl, from proteins (41). It is unknown whether circadian clock regulators are controlled by fatty acylation. Since fatty acid beta-oxidation is used to synthesize acetyl-CoA, circadian clock-controlled genes may sense NAD^+^ (redox carrier) levels or redox status *via* SIRT1 recruitment and connects to fatty acid metabolism *via* SIRT6 recruitment to control acetyl-CoA-levels. Aging involves greater risk of circadian rhythm disruption due to alteration of transcriptional regulation of clock-controlled genes (42). In a comparison of young (10 month) and old (22 month) mice, the addition of 3.2 g/L of the NAD^+^ precursor nicotinamide riboside reprogrammed and improved age-dependent disruption of circadian clock-controlled transcription in the liver, as monitored by BMAL1 ChIP sequencing (42).

## ADP-Ribosylation

NAD also plays roles in the transfer reaction of adenosine diphosphate (ADP). In ADP-ribosylation, one or more ADP-riboses from NAD^+^ are transferred to a protein by mono (ADP-ribose) or poly(ADP-ribose) polymerases (PARPs). Poly ADP-ribosylation (PARylation) regulates a variety of cellular processes including chromatin decondensation, transcription, DNA damage response, and mitosis (43). PARP1 binds the DNA damage site and recruits DNA repair molecules through PARylation (44). Inhibitors of PARP and poly(ADP-ribose) glycohydrolase (PARG) are used in cancer therapy (43). Interestingly, progestin promotes PARP1-mediated PAR generation, and PAR and its degradation to ADP-ribose (ADPR) are essential for increases in nuclear ATP levels in response to progestin in human breast cancer cells (45). Upon progestin treatment, ATP diffuses from mitochondria into the nucleus for around 10 minutes; meanwhile, enough NAD^+^ accumulates for the PAR synthesis reaction that occurs 10 minutes after supplying progestin. ADP-ribose from PAR degradation is used by ADP-sugar pyrophosphate nudix hydrolase 5 (NUDIX5), which synthesizes and maintains nuclear ATP used in chromatin remodeling to maintain progestin-responsive transcription (45). PARG also regulates chromatin remodeling of genes that respond to retinoic acid (46). Gene expression in response to nuclear hormones may use ATP and PAR to transiently obtain large amounts of energy.

## Nucleosides and RNA Editing

To escape from genome instability, damaged nucleotides are fixed by DNA repair pathways. Nucleosides in RNA are also edited. The editing of adenosine to inosine (A-to-I) in transcripts is catalyzed by adenosine deaminases (ADAs) (**Figure 1**) (47). This process frequently occurs at introns and 3’ untranslated regions (3’ UTRs). A greater frequency of A-to-I editing is found in micro RNA (miRNA) binding sites of target genes in tumor cells than in normal cells (47). The frequency of A-to-I editing by ADAs within miRNA is also greater in tumor cells than in normal cells (47). A single nucleotide polymorphism in the 3’ UTR of *MDM4*, which enables the binding of miR-191, leads to attenuation of tumor progression (48). However, pleural effusion ADA levels are significantly higher in malignant pleural mesothelioma patients than in healthy doners (49). Hence, changed expression of ADAs and miRNAs may be the consequences of tumorgenesis. Adenosine and inosine are products of *de novo* purine synthesis; inosine is also an intermediate of the purine salvage pathway (**Figure 1**). Alteration of A-to-I editing frequency may result from an imbalance in purine metabolism. ADA may adjust nucleoside pools by controlling inosine and miRNA. Measuring levels of other deaminases such as AMP deaminase and guanosine deaminase in addition to ADA levels may reveal that the nucleotide metabolic pathway is directly downstream of the tumor target. These measurements may also indicate the sensitivity of miRNA synthesis to each nucleotide. The 3’ UTR contains a poly(A) tail; a study showing how purine metabolism influences the poly(A) tail is also needed. The 5’ cap in mRNA consists of N^7^-methylguanine (N^7^meG), which connects to the first mRNA nucleotide and prevents mRNA degradation (50, 51). Since methylation of N^7^G requires SAMe, RNA processing may be affected by SAMe metabolism. Additional studies are needed to link pyrimidine metabolism to RNA synthesis.

## Discussion

Chromatin consumes metabolites. Examining the connections between metabolism, cellular events, epigenetic marks, and transcription will further reveal how epigenetics mirrors cellular metabolism. Monitoring chromatin status in the condition where the status of nucleotide biogenesis is modified will increase our understanding of the roles of epigenetics in one-carbon metabolism. The molecular linkages between chromatin modifiers and different nucleotides need further study.

## Author Contributions

TS wrote the manuscript. JW edited the manuscript. All authors contributed to the article and approved the submitted version.

## Funding

This research was supported by the Stowers Institute for Medical Research.

## Conflict of Interest

The authors declare that the research was conducted in the absence of any commercial or financial relationships that could be construed as a potential conflict of interest.

## Publisher’s Note

All claims expressed in this article are solely those of the authors and do not necessarily represent those of their affiliated organizations, or those of the publisher, the editors and the reviewers. Any product that may be evaluated in this article, or claim that may be made by its manufacturer, is not guaranteed or endorsed by the publisher.
